# The Therapeutic Applications of Liquid Air

**Published:** 1899-09

**Authors:** 


### THE THERAPEUTIC APPLICATIONS OF LIQUID AIR.

LIQUID air is a promising addition to the resources of medicine and surgery. Dr. A. Campbell White, of New York, has demonstrated its efficiency as a local anesthetic. The effect was painless and prompt, and many minor operations may be performed during anesthesia obtained by the refrigerating properties of liquid air applied in the form of a spray.

It has been found to be not only useful in this direction, but to possess a wide field of utility in the removal of obstinate superficial, even deep, lesions which have resisted the ordinary methods of treatment. It is said to afford great relief in neuralgia when sprayed over the affected area.

A case of lupus erythematosus has been brought to a cure without cicatrization under its use. Similarly the improvement in a case of epithelioma of the face has been most satisfactory. Nevus, carbuncles, furuncles, buboes, erysipelas, phagedenic chancroids, varicose and other ulcers of the leg have been treated with great satisfaction by this method.

Extensive experiments are being carried on by Dr. White at the Charity Hospital and the Vanderbilt Clinic, and a new field seems to have been opened up in medicine and surgery. The application is used in the form of a spray or a swab according to the effect desired. It is hoped that the rather exuberant claims made for it will be realized.

